# Evaluating Large Language Model (LLM) Performance on Established Breast Classification Systems

**DOI:** 10.3390/diagnostics14141491

**Published:** 2024-07-11

**Authors:** Syed Ali Haider, Sophia M. Pressman, Sahar Borna, Cesar A. Gomez-Cabello, Ajai Sehgal, Bradley C. Leibovich, Antonio Jorge Forte

**Affiliations:** 1 Division of Plastic Surgery, Mayo Clinic, Jacksonville, FL 32224, USA; 2 Center for Digital Health, Mayo Clinic, Rochester, MN 55905, USA; 3 Department of Urology, Mayo Clinic, Rochester, MN 55905, USA

**Keywords:** artificial intelligence, machine learning, large language models, plastic surgery, breast, capsular contracture, ectopic breast tissue, breast ptosis, gender-affirming mastectomy, gynecomastia

## Abstract

Medical researchers are increasingly utilizing advanced LLMs like ChatGPT-4 and Gemini to enhance diagnostic processes in the medical field. This research focuses on their ability to comprehend and apply complex medical classification systems for breast conditions, which can significantly aid plastic surgeons in making informed decisions for diagnosis and treatment, ultimately leading to improved patient outcomes. Fifty clinical scenarios were created to evaluate the classification accuracy of each LLM across five established breast-related classification systems. Scores from 0 to 2 were assigned to LLM responses to denote incorrect, partially correct, or completely correct classifications. Descriptive statistics were employed to compare the performances of ChatGPT-4 and Gemini. Gemini exhibited superior overall performance, achieving 98% accuracy compared to ChatGPT-4’s 71%. While both models performed well in the Baker classification for capsular contracture and UTSW classification for gynecomastia, Gemini consistently outperformed ChatGPT-4 in other systems, such as the Fischer Grade Classification for gender-affirming mastectomy, Kajava Classification for ectopic breast tissue, and Regnault Classification for breast ptosis. With further development, integrating LLMs into plastic surgery practice will likely enhance diagnostic support and decision making.

## 1. Introduction

To achieve consistent surgical outcomes, plastic surgeons strive to develop and implement classification systems. The variable nature of breast conditions has made it challenging to establish universally accepted classifications. Clearly, breast conditions necessitate a tailored surgical strategy [1,2,3]. Rapid advancements in clinical practice and the ever-growing arsenal of novel procedures require that surgeons constantly adapt their approach based on precise, individualized assessments.

Breast surgery demands a careful approach, balancing both aesthetic goals and functional needs due to the sensitive nature of the area [4]. Surgeons must navigate this balance with expertise, prioritizing procedures that enhance both the appearance and health of the breast. The first step in this case is to establish a correct diagnosis. This provides surgeons with essential tools for patient communication, surgical planning, and technique selection. It enables transparent discussions about anticipated cosmetic and functional outcomes, allowing surgeons to outline the potential risks and benefits of surgery. Standardized evaluation creates a common language for healthcare professionals, facilitating personalized and evidence-based care [5]. This knowledge also allows surgeons to tailor techniques, minimizing complications, reducing scarring, and lessening the likelihood of revision surgery.

As interest in AI grows, the medical research community is increasingly curious about its potential applications in healthcare [6]. The inherent complexity of breast conditions makes it an ideal area to explore the potential benefits of AI. AI-powered diagnostics in this area could lead to faster, more accurate diagnoses. They may even reveal subtle details that human experts might overlook [7]. Recent advancements, such as large language models (LLMs) like ChatGPT-4 and Gemini (formerly known as Bard), are currently being explored for their capabilities across various medical fields to enhance speed, improve diagnoses, and deliver efficient, scalable treatments [8,9]. Trained on extensive text data, these advanced AI systems can analyze vast information, identify patterns, and generate logical, coherent text, offering promising prospects for medical diagnostics.

Studies suggest that specialized LLMs outperform general models and baseline methods in various medical tasks, including diagnosis, decision making, and data analysis, and have the potential to transform medical care by improving accuracy and fostering interdisciplinary collaboration [10]. LLMs demonstrate significant potential within the medical field. They can enhance computer-aided diagnosis (CAD) systems for medical images [11], support personalized diagnosis and knowledge delivery [12], and generally mimic how healthcare professionals think [13]. This potential is particularly impactful in the context of telemedicine, where LLMs can enhance access to care, especially for patients in remote or underserved areas. This natural language processing capability could be crucial in telemedicine interactions, allowing for more nuanced communication between patients and providers. While LLMs show promise in enhancing telemedicine capabilities, it is crucial to acknowledge the inherent limitations of remote consultations. For instance, the absence of a physical examination can hinder accurate diagnoses, especially in specialties heavily reliant on physical examinations, such as orthopedics and spine surgery [14,15].

LLMs have shown promise in tasks like diagnostic reasoning [16]. They have shown remarkable progress in recent years in handling natural language processing across a wide range of healthcare applications [17]. Studies have demonstrated the surprising capability of LLM models like ChatGPT-3.5 and above to field basic questions related to severe medical conditions like cardiovascular disease and several cancers [18,19,20]. In a significant demonstration, ChatGPT achieved scores at or near passing levels on all segments of the United States Medical Licensing Examination (USMLE) [21]. On radiology board-style assessments, ChatGPT-3.5 inched closer to passing, while ChatGPT-4 demonstrably exceeded the knowledge benchmark [22,23].

To date, no study has yet evaluated the effectiveness of these LLMs in classifying breast diseases into different categories based on established classification criteria. This research investigates the baseline performance of publicly available LLMs, excluding retrieval-augmented generation (RAG). We will critically assess and compare the performance of two such models, ChatGPT-4 by OpenAI and Gemini by Google, in their ability to classify various breast diseases using established medical classification systems. Our analysis aims to ascertain each model’s proficiency in applying five renowned breast-related classification systems by leveraging a series of clinical vignettes representative of common and rare breast conditions. The study employs a comparative analysis approach, evaluating the LLMs’ accuracy and consistency in grading clinical vignettes across these classification systems. The implications of the findings, the promise of multimodal LLMs in plastic surgery, ethical considerations, and the limitations and challenges of the study are discussed, highlighting the potential of LLMs to support plastic surgeons in the classification of breast diseases and the future directions for research in this area. Through this comparative evaluation, our study seeks to illuminate the potential of LLMs to enhance diagnostic accuracy and decision making in plastic surgery and breast disease management, thereby contributing to improved patient care and outcomes.

## 2. Materials and Methods

Our study employed a comparative analysis approach to assess the diagnostic accuracy of LLMs in classifying common breast diseases. Five well-established breast disease classification systems from the existing literature were selected for evaluation: Baker classification for capsular contracture [24], Fischer classification for gender-affirming mastectomy [25], Kajava classification for ectopic breast tissue [3], Regnault classification for breast ptosis [26], and University of Texas Southwestern Medical Center (UTSW) classification for grading gynecomastia [27]. These classification systems provide a structured framework for diagnosing and categorizing breast conditions based on specific clinical criteria and findings.

Two state-of-the-art LLMs, ChatGPT-4 by OpenAI (San Francisco, CA, USA) and Gemini by Google (Mountain View, CA, USA), were chosen to participate in this assessment. Each model was independently tested using a set of clinical vignettes designed to represent typical presentations of the selected breast diseases. To ensure a comprehensive evaluation, 10 distinct clinical vignettes were crafted for each of the five breast diseases, culminating in a total dataset of 50 vignettes. The vignettes were developed to include a range of clinical features and scenarios that clinicians might encounter, thereby challenging the LLMs’ ability to accurately identify the appropriate disease grade based on the information provided.

**Figure 1:**
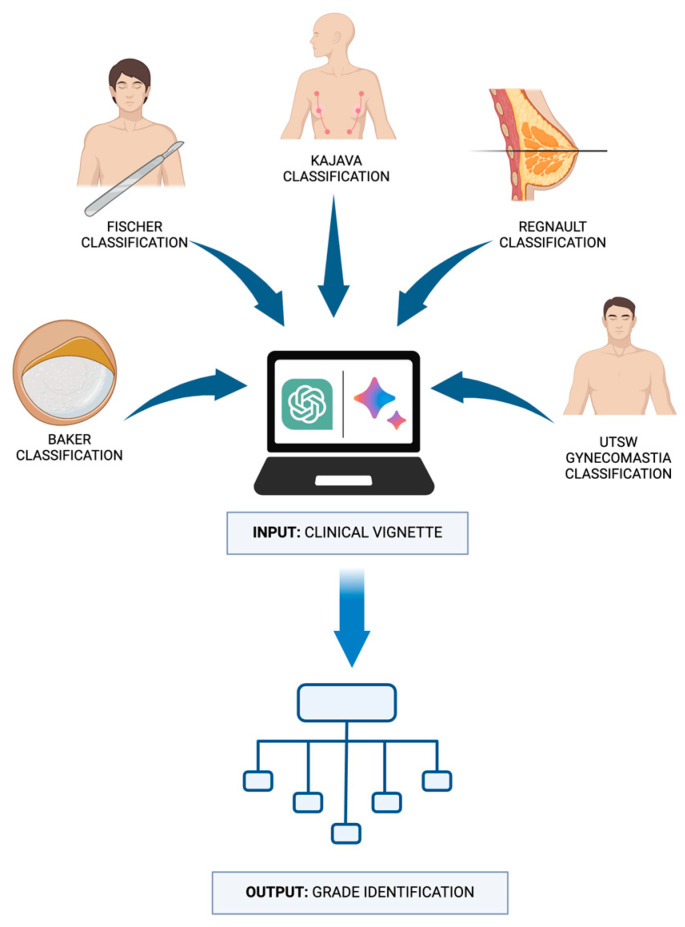
Evaluation of ChatGPT and Gemini in identifying breast disease classification based on clinical vignettes.

The vignettes were presented to each LLM, requiring the models to interpret the clinical information and make classification decisions based solely on the content of the vignettes. The LLMs’ responses were then recorded and compared against the correct classifications according to the established systems to determine their accuracy. This design aimed to simulate a real-world clinical decision-making process, evaluating the LLMs’ capability to correctly categorize breast diseases in a manner akin to a healthcare professional’s approach (Figure 1). 

### 2.1. Selection of Classification System and Rationale

The classification systems used in this study are well-documented in publicly available NIH articles. These systems include the following.

#### 2.1.1. Baker Classification for Capsular Contracture

The Baker classification system assesses the severity of capsular contracture, a potential postoperative complication following breast augmentation [24]. This condition results from the development of scar tissue around the breast implants, leading to firmness, distortion, or discomfort in the breasts. The system distinguishes four levels of severity for capsular contracture as follows (Table 1).

The surgical plan for a mild capsular contracture, which will have scarring around a breast implant, will be vastly different from a severe one. Assessment often relies on physical examination to feel for firmness and wrinkling, which can be subjective and vary depending on the surgeon’s experience [28]. Imaging tests may only sometimes be definitive [29]. Depending on the grade, treatment could range from observation and monitoring to implant removal and capsulectomy (scar tissue removal). Early detection of mild contractures can be difficult. Additionally, differentiating capsular contracture from other implant complications like implant rupture can be challenging [30].

#### 2.1.2. Fischer Grade Classification for Gender-Affirming Mastectomy

The Fischer Grade Classification guides the choice of surgical method in gender-affirming mastectomy and helps attain visually appealing results with minimal scarring on the chest wall [25]. It is guided by specific attributes of the breasts, including the size of the breasts, the amount and elasticity of the skin, the dimensions of the chest, and the size and location of the nipple–areola complex as follows (Table 2):

This classification system empowers surgeons to select the most suitable mastectomy technique for achieving an optimal masculine chest appearance with minimal scarring. By predicting potential outcomes for different approaches, the system also minimizes complication risks and the need for revision surgery.

#### 2.1.3. Kajava Classification for Ectopic Breast Tissue

The Kajava classification for ectopic breast tissue categorizes supernumerary or accessory breast tissues based on their anatomical components [3]. This classification system identifies eight different classes (Table 3).

The Kajava classification helps guide treatment decisions. For instance, symptomatic accessory breast tissue with glandular components may require surgical removal. Ectopic breast tissue can present in various locations and may mimic other conditions, making diagnosis difficult, especially if it is deep within the chest tissue [31,32].

#### 2.1.4. Regnault Classification for Breast Ptosis

The Regnault classification for breast ptosis categorizes the degree of sagging based on the position of the nipple–areolar complex (NAC) relative to the inframammary fold (IMF) and the distribution of breast tissue [26]. It is crucial for planning surgical procedures, such as breast lifts or augmentations, to ensure the desired aesthetic outcomes and patient satisfaction (Table 4).

Several challenges are posed in accurately identifying breast ptosis grades. Firstly, the assessment can be somewhat subjective, as it relies on a visual evaluation of the breast in relation to the inframammary fold (IMF). Variations in body shape, breast size, and skin elasticity between individuals can make the grading process less precise. Additionally, factors like age and body mass index (BMI) can influence the natural position of the breast and nipple. Correctly grading ptosis helps to determine the most appropriate surgical approach if a breast lift (mastopexy) is desired.

#### 2.1.5. The University of Texas Southwestern Medical Center (UTSW) Classification for Gynecomastia

The UTSW Classification for Gynecomastia grades gynecomastia based on the extent of breast enlargement and skin excess. This classification helps surgeons tailor their approach to each patient’s specific needs, ensuring optimal cosmetic outcomes and patient satisfaction (Table 5).

Classifying gynecomastia (enlarged male breasts) helps determine the cause and the most effective surgical approach (liposuction, gland removal, or a combination of both). Distinguishing between fatty and glandular tissue enlargement can be challenging on physical examination alone.

### 2.2. Creation of Clinical Vignettes

**Figure 2:**
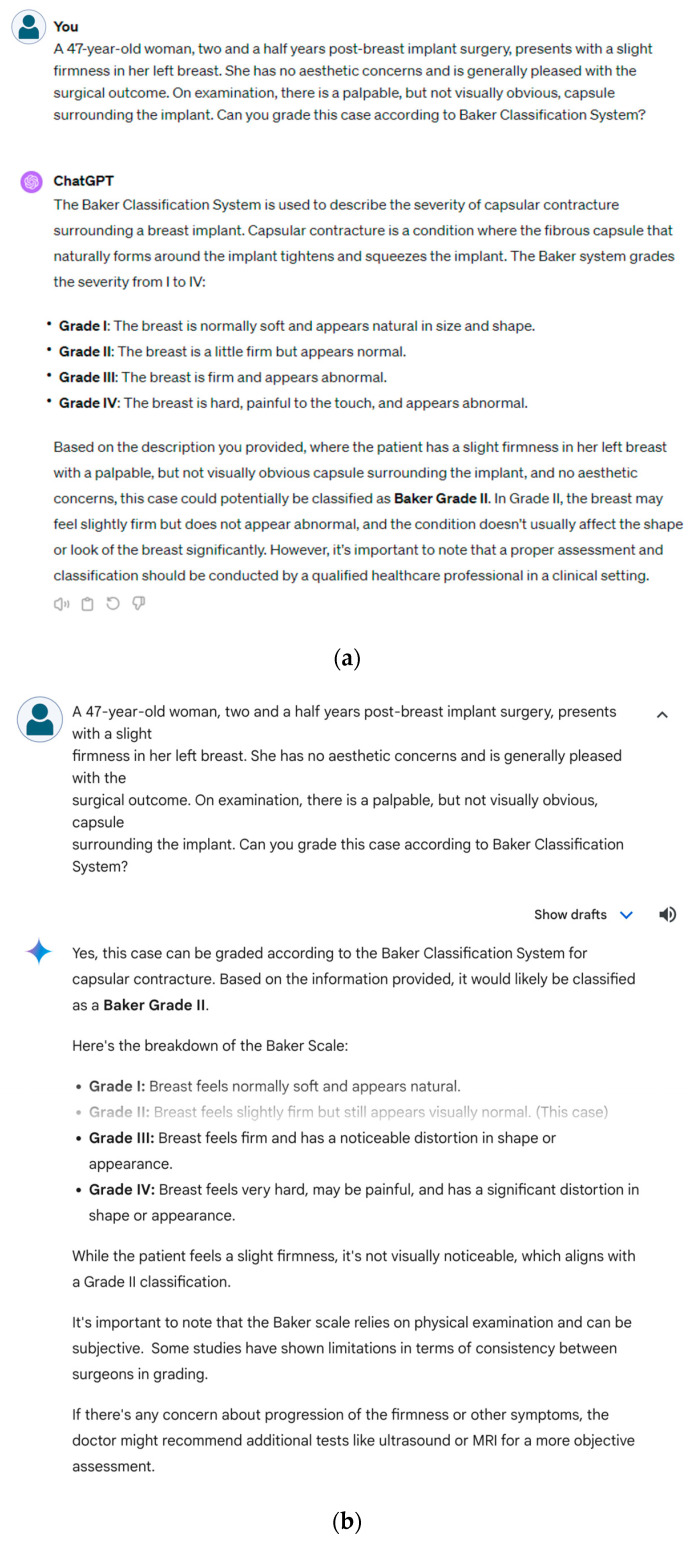
Baker classification grading examples from ChatGPT (**a**) and Gemini (**b**) for a capsular contracture case.

For each classification system, clinical vignettes were developed systematically. Each vignette reflected the diverse presentations of the selected breast condition. A detailed review of the chosen classification systems was conducted that focused on the specific criteria and definitions of breast disease. This review served as a foundation for constructing vignettes. For each of the five disease classification systems, 10 vignettes were designed. Within each classification system, at least one vignette was dedicated to discussing at least one specific grade, ensuring comprehensive coverage across all grades within each system. The vignettes encapsulated the key features and symptoms of clinical presentation associated with the grade with the incorporation of realistic details. Each fictional patient was assigned an age, sex, physical examination findings, and clinical symptoms. All 10 vignettes for the Baker, Fischer, and Regnault classifications were female patients. For the UTSW gynecomastia classification, all 10 patients were male. For the Kajava classification of ectopic breast tissue, 3 cases were male, and 7 cases were female. Patient ages were evenly distributed within each classification to represent a wide patient population: Baker (38.6 ± 7.5 years), Fischer (29.8 ± 7.7 years), Regnault (41.4 ± 8.1 years), UTSW (28.6 ± 8.4 years), and Kajava (29.4 ± 14.6 years). The vignettes were presented to ChatGPT-4 (Figure 2a) and Gemini (Figure 2b) along with this specific prompt:

“*Can you grade this case according to [specific classification] system?*”

This standardized question required the LLMs to apply their understanding of the classification criteria to the information provided in the vignette and make a reasoned judgment regarding the appropriate grade or stage. The responses from the LLMs were recorded for subsequent comparison with the original grades based on the established systems.

### 2.3. Evaluation Criteria

For each classification, we presented the LLMs with 10 clinical vignette-style questions designed to reflect the various grades of breast diseases, serving as the ground truth against which the LLMs’ responses were compared.

The LLMs’ responses were evaluated using a rating scale of 0 to 2 as follows:

- 0: Inaccurate;
- 1: Partially accurate (at least one correct answer among multiple options);
- 2: Fully accurate.

With 10 vignettes per classification system, the maximum possible score for each system was 20. Accuracy was calculated as a percentage of this maximum score. For instance, an LLM achieving a score of 16 (8 fully accurate responses out of 10) would have an accuracy of 80%. To assess and compare the LLMs’ performance, we calculated the mean, median, mode, and standard deviation of scores for each classification. The F1 score, a balanced metric incorporating precision and recall, was calculated for each model across five breast-related classification systems. A score of 1–2 was considered as positive and a score of 0 was considered as negative. These metrics provided insights into the consistency and variability of LLM performance across breast disease classifications.

## 3. Results

The results of the experiment reveal Gemini’s clear advantage over ChatGPT-4 in several key metrics.

### 3.1. Overall Performance

Gemini boasted a remarkable overall accuracy of 98% compared to ChatGPT’s 71%. Further, Gemini’s average score per response was 1.96, exceeding ChatGPT’s mean of 1.42, which shows that Gemini was more likely to classify the grade completely correctly. Central tendency measures, including median and mode scores, also favored Gemini. Both metrics were equal to 2.0 for Gemini, while ChatGPT fell behind with an average median of 1.6 and an average mode of 1.4 (Table 6).

### 3.2. Cross-Classification Performance

Both ChatGPT and Gemini performed well on the Baker classification test for capsular contractures, with average mean scores of 1.8 and 2.0, respectively, and accuracy rates exceeding 90%. Notably, both models achieved a perfect accuracy (100%) when classifying gynecomastia using the UTSW system. However, a clear difference emerged in the Fischer Grade Classification. Here, Gemini demonstrated significantly better performance, achieving a perfect accuracy (100%) with a mean average of 2.0, while ChatGPT 4 only managed a mean average of 0.9 and a 45% accuracy rate. This trend continued across other classification systems. In the Kajava classification for ectopic breast tissue, Gemini maintained its superiority with 100% accuracy and an average mean of 2.0, compared to ChatGPT 4’s 70% accuracy and average mean of 1.4. Similarly, Gemini excelled in the Regnault classification for breast ptosis with a mean score of 1.8 and 90% accuracy, surpassing ChatGPT 4’s average of 1.0 and 50% accuracy. These results highlight Gemini’s consistent strength across various classification tasks, particularly in medical contexts, when compared to ChatGPT 4.

ChatGPT demonstrates significantly higher response variability, with a standard deviation of 0.68 compared to Gemini’s 0.13. This indicates that ChatGPT’s responses were less consistent, while Gemini’s lower standard deviation implies greater reliability.

Confusion matrices, along with precision, recall, and F1 score for each classification system, are presented below in Table 7a–e. The overview of the performance of both LLMs are provided in Table 8.

## 4. Discussion

Patient variability in breast disorders hinders the development of a standardized classification system. This subjectivity arises from inherent differences in how breast tissue presents itself among individuals. First, breast tissue appearance varies naturally from person to person. This is due to factors such as genetics, hormones, and age [33,34]. Hormonal shifts throughout a woman’s life, from menstruation and pregnancy to menopause, can cause noticeable changes in the look and feel of her breasts [35]. Second, the clinical techniques used to diagnose and evaluate breast conditions can be imprecise, often relying on visual inspection and palpation [36]. Such subjective methods can lead to inconsistencies in how conditions are assessed and graded. Finally, the goals of treatment in plastic surgery may differ from those in other medical fields. The focus may be on improving cosmetic appearance rather than solely on addressing a pathological condition. This lack of standardization can be problematic, as without clear classifications, different surgeons may diagnose the same condition in various ways. This can be confusing for patients and hinder surgeons from comparing results. Without universally implemented standardized classification systems for breast conditions in plastic surgery, conducting large-scale studies and comparing results across institutions becomes highly challenging.

This proof-of-concept study reveals the considerable potential of LLMs to revolutionize the classification of breast diseases within plastic surgery. These results demonstrate that Gemini outperformed ChatGPT in terms of accuracy and consistency. This is supported by Gemini’s higher mean and median scores, as well as its lower standard deviation. Both LLMs effectively offered timely and appropriate advice for diagnosing breast diseases, but Gemini consistently proved superior. Even though diagnostic performance within different breast classification systems varied, Gemini achieved an impressive overall accuracy of 98%, significantly surpassing ChatGPT-4’s 71%. This advancement highlights the remarkable ability of LLMs to understand and apply complex medical classifications. The difference was particularly evident in the Fischer Grade Classification system, where Gemini achieved perfect accuracy (100%), while ChatGPT-4 lagged behind at 45%. Gemini maintained its advantage across the Kajava and Regnault classification systems. This high accuracy demonstrates that Gemini was trained on more complete information for ectopic breast tissue and breast ptosis characteristics. Interestingly, both models performed equally well compared with the UTSW gynecomastia classification, both achieving 100% accuracy.

Gemini’s superior performance can likely be attributed to several factors. First, Gemini was trained on a larger and more up-to-date dataset, particularly in the medical domain. Google has been working with specialized medical domain models like MED-PALM 2 [37] and Med-Gemini [38], and it is conceivable that Google leverages the knowledge and advancements from these specialized AI models to enhance Gemini’s capabilities in the medical field. Additionally, Gemini may have undergone more extensive fine-tuning on medical literature and datasets related to breast diseases. The black-box nature of large language models poses a challenge in understanding how these models arrive at their conclusions, which is crucial for building trust and ensuring their safe integration into clinical practice. To address the black-box nature of these models and improve their explainability, it is crucial to implement more transparent models and develop post hoc methods for the explanation that can elaborate on the outputs of black-box models [39]. Continuous clinical trials testing the performance of these models and comparing their performance to established clinical guidelines can help validate their classification decisions and ensure alignment with clinical benchmarks.

By focusing on explainability, transparency, and rigorous clinical testing, we can build trust in the use of LLMs in clinical practice and gain a deeper understanding of their decision-making processes. This will be essential for the safe and effective integration of these powerful tools into healthcare settings. LLMs may be ready for clinical testing under physician supervision. Gemini’s superior accuracy compared to ChatGPT-4 showcases the rapid progress of AI in healthcare. Despite its later release (March 2023) compared to ChatGPT-4 (late 2022), Gemini’s performance is a testament to the ongoing advancements in this field. Their remarkable accuracy has far-reaching implications for clinical decision making. By integrating LLMs into the process of grading breast conditions, clinicians can reduce diagnostic errors, ultimately improving patient outcomes significantly. LLMs can be trained to recognize and account for individual variations, ultimately aiding in the development of new classification criteria that could potentially address the lack of universally accepted breast disease classifications. Additionally, training LLMs on extensive real-life clinical data could address their shortcomings, like hallucinations or false information [40]. These clinical data should account for patient variability and subtle nuances that are lacking in textbook cases. Exposure to documented misdiagnoses and challenging cases allows LLMs to analyze the patterns and potential decision points that led to errors. By recognizing these patterns, the model can learn to avoid the same mistakes, reducing the occurrence of hallucinations and delivering more reliable information [41]. Real-life data allow LLMs to learn about comorbidities and other factors that can influence breast tissue. By recognizing these potential confounders, LLMs can refine their classifications for greater accuracy.

### 4.1. The Promise of Multimodal LLMs

Gemini’s precision holds promise as a second-opinion tool in medical diagnostics, potentially assisting in identifying nuanced distinctions between disease grades. This study has significant implications for the future integration of AI in surgery. Building on this success, further research could explore AI’s role in addressing higher-order questions pertaining to disease management and potential complications. The next frontier for AI-driven diagnostics lies in multimodal LLMs that can integrate diverse forms of data, including images, videos, voice, and text [42]. By merging insights from various sources, these models obtain a more complete picture of the patient [43]. Gemini itself is a multimodal AI system that is offered in Ultra, Pro, and Nano variants [44]. As Google’s largest and most intelligent Gemini model, Gemini Ultra offers superior performance in natural language processing and complex tasks. It is also the most resource-intensive version. Gemini Pro is a versatile mid-sized model that balances performance and efficiency. It is perfect for everyday tasks and seamless integration into various applications. Gemini Nano offers a compact version of Google’s Gemini language model, prioritizing computational efficiency and rapid response times. This optimization makes it suitable for low-power devices or real-time scenarios, potentially at the cost of some complex functionalities available in larger models. Gemini’s multimodality allows it to understand, process, and synthesize information across diverse modalities, including textual data with PDF support, images, audio, video, and computer code. The model exhibits generative capabilities in both text and image domains [45].

A potential use case is a multimodal LLM analyzing a mammogram alongside a patient’s medical history, and even voice recordings from consultations could provide a more comprehensive diagnosis. The integration of generative AI with LLMs could revolutionize patient communication and surgical planning. Generative AI, adept at creating realistic images and videos, could be harnessed to generate personalized visualizations of surgical procedures [46,47,48]. For a patient undergoing gender-affirming surgery, the LLM could factor in the chosen surgical grade and utilize generative AI to create a video outlining the surgical steps and potential final results. This approach would provide invaluable pre-operative information, allowing patients to visualize the surgical journey and its outcome. Similarly, in procedures like gynecomastia surgery, generative AI could factor in the grade of gynecomastia and create personalized visualizations of achievable results, fostering realistic expectations and potentially reducing anxiety for patients. This synergy between LLMs and generative AI has the potential to empower patients, fostering informed decision making and a more positive surgical experience. Such a holistic approach leads to more robust decision making, potentially improving patient outcomes. Additionally, studies have demonstrated that patients feel significantly more at ease when surgeons dedicate ample time to pre- and post-operative consultations [49]. These interactions build trust, address concerns, and ensure the patient feels informed and supported. If multimodal LLMs can expedite the technical aspects of diagnosis and classification, they would essentially give surgeons the flexibility to build their connection with the patient. This freed-up time could be reinvested into the patient–doctor relationship. Surgeons would have a greater capacity for meaningful consultations, personalized counseling, and providing the emotional support that is crucial throughout a patient’s surgical journey [50].

### 4.2. Expanding the Role of LLMs in Plastic Surgery

The success of LLMs in classifying breast diseases within plastic surgery signals a broader opportunity to leverage these advanced AI tools for enhanced education and specialized diagnostic applications. Integrating LLMs into the training of medical students and residents offers a revolutionary approach to learning [51]. Through simulated, interactive clinical experiences, learners can refine their diagnostic reasoning, receive tailored feedback, and develop the critical decision-making skills essential for real-world practice [51]. LLMs hold immense potential to transform diagnostic accuracy across various subspecialties within surgery. In craniofacial surgery, where complex anatomical anomalies and reconstructions are common, LLMs could provide support. These models could classify congenital conditions, propose surgical interventions, and predict potential outcomes informed by their vast datasets of medical knowledge. Similarly, in hand surgery, functional outcomes are of paramount importance. LLMs could analyze anatomical details and functional assessments to classify hand conditions, aiding surgeons in selecting procedures that maximize patient recovery and satisfaction. Within aesthetic surgery, LLMs have the potential to become sophisticated predictive tools. By simulating the results of different procedures, they can assist surgeons in personalizing their approach, aligning results with patient expectations, and leading to improved aesthetic outcomes. Continuous training and refinement of these models are crucial, and collaborative efforts between AI researchers and plastic surgeons will help ensure these tools effectively address the complexities of real-world clinical scenarios.

### 4.3. Ethical Considerations and Patient Privacy

While the potential of advanced language models in healthcare is undeniable, prioritizing ethical concerns and safeguarding patient privacy remains of utmost importance [52,53]. The inherent diversity of patient presentations necessitates adaptable models that avoid perpetuating health inequities. The sometimes-opaque nature of these models’ decision-making processes highlights the need for transparency and accountability mechanisms [54]. As the physician–patient relationship evolves in this technology-driven landscape, we must guard against over-relying on these tools and uphold the value of clinical judgment. Protecting sensitive patient data is paramount, and rigorous adherence to regulations such as HIPAA (Health Insurance Portability and Accountability Act) is essential. HIPAA provides stringent standards for handling Protected Health Information (PHI), such as data encryption, controlled access, and detailed record keeping [55]. The first crucial step to safeguard patient privacy is data anonymization, which involves scrubbing all patient data used for training and testing of any direct patient identifiers [56]. For rare diseases, even indirect identifiers and dates of procedures should be altered to maintain confidentiality. Equally important is the secure storage of encrypted data. Patient data and LLM outputs should be stored on secure, encrypted servers that protect the information even during transmission between systems [57]. Developers of LLMs, including data providers and healthcare teams, must establish clear data use agreements that outline measures to be taken in case of a privacy breach.

Another essential component is obtaining informed consent from patients before their data are used for LLM training. Informed consent ensures that patients are fully aware of how their data will be used, the purpose of the data collection, and the methods employed to analyze their data [58]. This process should also include a clear explanation of the measures in place to protect patient privacy. Fostering patient understanding of how these models are used, alongside the safeguards in place to protect their data, is crucial for building trust.

Furthermore, when collecting patient data, it is crucial to adhere to the principle of minimum collection, ensuring that only the data necessary for model training are gathered [59]. By upholding these principles, we ensure that LLMs can improve patient care while respecting their privacy and rights, laying the groundwork for ethical and responsible integration of technology into healthcare.

## 5. Limitations and Challenges

Unlike the vignettes designed to reflect specific disease grade descriptions, clinical scenarios are rarely presented in textbook presentations. The study did not explicitly focus on potential biases in the LLMs’ performance related to patient demographics. We used a basic prompt for the LLMs, which may not be representative of the diverse patient demographics encountered in clinical practice. Future studies should consider using prompts that better reflect the variety of patient characteristics and presentations to assess the LLMs’ performance more accurately across different patient populations. LLMs like ChatGPT-4 and Gemini are trained on a huge corpus of data from various sources, which may introduce biases stemming from inherent biases present in the training data. These biases could potentially impact the models’ performance when classifying breast diseases across different patient populations. Future research should focus on identifying and mitigating such biases to ensure equitable and accurate diagnosis for all patients, regardless of their demographic characteristics. This could involve analyzing the LLMs’ performance across diverse patient groups, examining the training data for potential sources of bias, and developing strategies to reduce the impact of these biases on the models’ outputs.

Moreover, the focus on first-order questions solely aimed at diagnosis may not fully explore the capabilities of the LLMs in the complexities of actual clinical situations. This study evaluated a limited scope of five breast disease classifications, potentially not addressing the broader range of concerns within breast/plastic surgery. For example, future research could expand to include a larger set of clinical vignettes and rare breast diseases, benign tumors, and other surgical conditions to better represent the diversity of clinical presentations. However, the focus on common breast diseases and clinical vignettes provided a valuable starting point for evaluating LLM accuracy and precision in breast disease classification.

The comparison was restricted to GPT-4 (the paid, most advanced OpenAI version) and the freely available Gemini model. Further research could explore GPT-4 against more advanced Gemini versions or other chatbots such as Microsoft’s Copilot (Bing AI), Claude, or even the basic GPT 3.5 [60,61]. It is also essential to investigate the reasons for the specific types of errors generated by different LLMs. This analysis helps identify areas where traditional methods still maintain an advantage over LLMs.

## 6. Conclusions

This study demonstrates the potential of LLMs, particularly Gemini, to support plastic surgeons in the classification of breast diseases. Gemini consistently outperformed ChatGPT-4, exhibiting superior accuracy across most of the investigated systems. These findings suggest that the integration of LLMs into plastic surgery practice has the potential to significantly improve diagnostic accuracy and streamline decision-making processes. Exploring the application of LLMs in plastic surgery signifies a paradigm shift in personalized patient care. The enhanced diagnostic precision and decision-making support provided by models like Gemini can lead to more tailored and effective treatment plans, ultimately elevating patient outcomes and satisfaction. As technology and human expertise combine, patients will experience a new era of healthcare defined by superior diagnostic capabilities powered by advanced AI, which empowers surgeons to anticipate and manage risks with greater precision, allowing them to personalize interventions for each patient’s unique circumstances [62]. LLMs can learn from diverse datasets and adapt to the complex nature of breast conditions. This capability could lead to the development of more sophisticated and widely applicable classification systems for these conditions.

Furthermore, integrating LLMs into clinical practice extends beyond the immediate benefits of improved diagnostics. As these models evolve and learn from a vast array of clinical scenarios, they can become invaluable resources for ongoing medical education and training. Beyond serving as repositories of vast medical knowledge, LLMs can become active participants in the learning process. LLMs capable of generating highly realistic clinical vignettes can accurately reflect the subtle presentations of breast diseases. These vignettes, incorporating specific classification criteria, could challenge students and residents to apply their knowledge, test their diagnostic reasoning, and receive immediate, personalized feedback from the LLM. This interactive format creates a simulated clinical environment, allowing learners to practice and refine their skills before encountering real patients. Moreover, as LLMs continuously adapt to the latest advancements, they provide a seamless way for practitioners to stay abreast of the most current research and guidelines in breast disease management, fostering lifelong learning. Since this experiment used publicly available general LLMs, further research is warranted to assess the efficacy of retrieval-augmented generation (RAG) techniques for breast disease classification. The true value of LLMs lies in their ability to augment human expertise, leading to more informed decision making and a deeper understanding of patient needs, thus shaping the future of surgery and patient care.

## Figures and Tables

**Table 1:** Baker classification for capsular contracture.

Baker Grade	Description
I	Normal, soft, nonpalpable implant
II	Palpable, minimally firm to touch, not visible
III	Visible, easily palpated, and moderately firm
IV	Painful, hard, and breast distorted

**Table 2:** Fischer Grade Classification for gender-affirming mastectomy.

Fischer Grade	Breast Size	Skin Laxity	Surgical Approach
I	Small	No laxity	Circum-areolar incision technique preferred
IIA	Moderate	Less/more defined	Circum-areolar incision technique preferred
IIB	Moderate	More variable	Free nipple graft technique
III	Large	Moderate to significant	Free nipple graft technique
IV	Deflated Appearance	Significant	Free nipple graft technique

**Table 3:** Kajava classification for ectopic breast tissue.

Kajava Grade	Tissue Present
I	Nipple, areola, glandular (polymastia)
II	Nipple and glandular
III	Areola and glandular
IV	Glandular only
V	Nipple and areola (pseudomamma)
VI	Nipple only (polythelia)
VII	Areola only (polythelia areolar)
VIII	Patch of hair (polythelia pilosa)

**Table 4:** Regnault classification for breast ptosis.

Regnault Class	Description
Pseudoptosis/Glandular Ptosis	NAC * at/above IMF **, most tissue below.
Grade I Ptosis (Mild)	NAC at IMF level.
Grade II Ptosis (Moderate)	NAC below IMF, above lowest tissue.
Grade III Ptosis (Severe)	NAC well below IMF, at lowest tissue.

* NAC: nipple–areolar complex, ** IMF: inframammary fold.

**Table 5:** UTSW classification for gynecomastia.

UTSW Grade	Description
I	Mild hypertrophy (<250 g of breast tissue), no ptosis
I.A.	Mainly glandular
I.B.	Mainly fibrous
II	Mild (250 g) to Moderate (500 g) Hypertrophy, no ptosis
IIA	Mainly glandular
IIB	Mainly fibrous
III	Severe (>500 g) Hypertrophy of breast tissue, +grade I ptosis
IV	Severe (>500 g) Hypertrophy, + grade II/III ptosis

**Table 6:** Overall performance by LLMs.

**Mean**	**ChatGPT-4**	**Gemini**
Accuracy	71%	98%
Total score	14.2	19.6
Score per response	1.42	1.96
Median	1.6	2.0
Mode	1.4	2.0
Standard Deviation (SD)	0.68	0.13

**Table 7:** (**a**) Baker classification. (**b**) Fischer Grade Classification. (**c**) Kajava classification. (**d**) Regnault classification. (**e**) UTSW classification.

	ChatGPT			Gemini	
	Predicted+	Predicted−		Predicted+	Predicted−
(**a**)					
**Actual+**	9	1	**Actual+**	10	0
**Actual−**	0	0	**Actual−**	0	0
*Precision*	1		*Precision*	1	
*Recall*	0.9		*Recall*	1	
*F1 Score*	0.95		*F1 Score*	1.00	
(**b**)					
**Actual+**	7	3	**Actual+**	10	0
**Actual−**	0	0	**Actual−**	0	0
*Precision*	1		*Precision*	1	
*Recall*	0.7		*Recall*	1	
*F1 Score*	0.82		*F1 Score*	1.00	
(**c**)					
**Actual+**	7	3	**Actual+**	10	0
**Actual−**	0	0	**Actual−**	0	0
*Precision*	1		*Precision*	1	
*Recall*	0.7		*Recall*	1	
*F1 Score*	0.82		*F1 Score*	1.00	
(**d**)					
**Actual+**	5	4	**Actual+**	9	1
**Actual−**	1	0	**Actual−**	0	0
*Precision*	0.83		*Precision*	1	
*Recall*	0.56		*Recall*	0.9	
*F1 Score*	0.67		*F1 Score*	0.95	
(**e**)					
**Actual+**	10	0	**Actual+**	10	0
**Actual−**	0	0	**Actual−**	0	0
*Precision*	1		*Precision*	1	
*Recall*	1		*Recall*	1	
*F1 Score*	1.00		*F1 Score*	1.00	

**Table 8:** Overview of the results.

**Classification System**	**LLM**	**Accuracy %**	**Total Score**	**Mean**	**Median**	**Mode**	**SD**	**F1-Score**
Baker Classification	ChatGPT-4	90	18	1.8	2	2	0.63	0.95
	Gemini	100	20	2	2	2	0.00	1.00
Fischer Grade Classification	ChatGPT-4	45	9	0.9	1	1	0.74	0.82
	Gemini	100	20	2	2	2	0.00	1.00
Kajava Classification	ChatGPT-4	70	14	1.4	2	2	0.97	0.82
	Gemini	100	20	2	2	2	0.00	1.00
Regnault Classification	ChatGPT-4	50	10	1	1	0	1.05	0.67
	Gemini	90	18	1.8	2	2	0.63	0.95
UTSW Gynecomastia	ChatGPT-4	100	20	2	2	2	0.00	1.00
	Gemini	100	20	2	2	2	0.00	1.00

## Data Availability

The raw data supporting the conclusions of this article will be made available by the authors on request.
